# Motional Fock states for quantum-enhanced amplitude and phase measurements with trapped ions

**DOI:** 10.1038/s41467-019-10576-4

**Published:** 2019-07-02

**Authors:** Fabian Wolf, Chunyan Shi, Jan C. Heip, Manuel Gessner, Luca Pezzè, Augusto Smerzi, Marius Schulte, Klemens Hammerer, Piet O. Schmidt

**Affiliations:** 1 Physikalisch-Technische Bundesanstalt, Bundesallee 100, 38116 Braunschweig, Germany; 2 QSTAR, INO-CNR and LENS, Largo Enrico Fermi 2, I-50125 Firenze, Italy; 3 Institute for Theoretical Physics, Institute for Gravitational Physics (Albert Einstein Institute), Leibniz Universität Hannover, Appelstrasse 2, 30167 Hannover, Germany; 4 Institut für Quantenoptik, Leibniz Universität Hannover, Welfengarten 1, 30167 Hannover, Germany; 5 Present Address: Département de Physique, École Normale Supérieure, PSL Université, CNRS, 24 Rue Lhomond, 75005 Paris, France

**Keywords:** Atomic and molecular interactions with photons, Quantum metrology

## Abstract

The quantum noise of the vacuum limits the achievable sensitivity of quantum sensors. In non-classical measurement schemes the noise can be reduced to overcome this limitation. However, schemes based on squeezed or Schrödinger cat states require alignment of the relative phase between the measured interaction and the non-classical quantum state. Here we present two measurement schemes on a trapped ion prepared in a motional Fock state for displacement and frequency metrology that are insensitive to this phase. The achieved statistical uncertainty is below the standard quantum limit set by quantum vacuum fluctuations, enabling applications in spectroscopy and mass measurements.

## Abstract (web summary)

Quantum metrology allows surpassing the standard quantum limit, but methods relying on squeezing require to know the orientation of the squeezed quadrature with respect to the signal. Here, instead, the authors propose a phase-insensitive Fock-state-based protocol, and demonstrate it using trapped ions.

## Introduction

Non-vanishing fluctuations of the vacuum state are a salient feature of quantum theory. These fluctuations fundamentally limit the precision of quantum sensors. Advances in the ability to control quantum systems together with the suppression of classical noise originating from technical imperfections, has led to the emergence of sensors, such as optical clocks^1^, gravitational wave detectors^2^, matter-wave interferometers, magnetometers^3^, and optomechanical systems^4^, that approach measurement sensitivities where the effect of quantum fluctuations sets a fundamental limit, the so called standard quantum limit (SQL). For more than 30 years it has been known that certain types of non-classical states can reduce the effect of quantum noise and thus enhance the sensitivity of measurement devices beyond the classical limit^5^. Taking advantage of this sub-SQL sensitivity requires not only the preparation of the non-classical state with high fidelity, but also the prevention of signal loss in the entire measurement protocol. This has been achieved e.g., with squeezed states and Schrödinger-cat or N00N states in interferometric settings^6–9^. A common restriction of these types of non-classical states is the need for control over the relative phase between the state creation and the measurement interaction^10,11^. Lack of control can lead to an amplification of noise and reduces the sensitivity of the device. In a phase-space picture, squeezing along the displacement direction enhances the sensitivity for amplitude measurements, but weakens the sensitivity for phase measurements.

Here we experimentally demonstrate a quantum metrological paradigm based on phase-insensitive motional Fock states^12^ of a trapped ion, with applications in frequency metrology and displacement detection. More specifically, we present sub-SQL measurements of amplitude and phase of the motional state of a trapped ion using the same motional Fock state. This is enabled by the implementation of a measurement scheme that allows direct detection of individual Fock state populations (see Methods). The measurement apparatus is operated in two different experimental settings, each probing displacements in one of two orthogonal quadrature components with sensitivities beyond the SQL using the same initial quantum state. Firstly, the amplitude of the ion’s oscillation is varied and the phase is kept constant, which realizes a displacement or force sensor^13–15^. Secondly, the Fock state is displaced with a fixed amplitude in a Ramsey-like interferometry sequence to measure the phase of the ion’s oscillation, which implements a measurement of the oscillation frequency of the ion in the trap. In both measurements, classical preparation and detection noise are sufficiently small to preserve the quantum gain in a full metrological protocol. Furthermore, we prove that Fock states are optimal for sensing displacements with unknown phase.

## Results

### Experimental apparatus

The experiments are performed with a single ^25^Mg^+^ ion confined in a linear Paul trap. Excited motional Fock states are created starting from the motional and electronic ground state^16^, through a sequence of laser-driven blue and red sideband pulses that each add a quantum of motion while changing the internal state of the ion^17,18^. A calibrated displacement $\hat D(\alpha ) = {\mathrm{exp}}\left( {\alpha \hat a^\dagger - \alpha ^ \ast \hat a} \right)$ is implemented by exposing the ion to an electric field oscillating at the trapping frequency of *ω*_*z*_ = 2*π* × 1.89 MHz. The displacement amplitude |*α*| can be controlled through the modulation time *t*_F_ (see Methods for more details). It is measured by mapping the overlap between initial and displaced state onto the atomic qubit (|↑〉, |↓〉, encoded in two hyperfine states of the ^2^S_1/2_ electronic ground state of ^25^Mg^+^), where state-readout is performed using state dependent fluorescence^19^. The mapping process is implemented by a sequence of sideband rapid adiabatic passage (RAP)^20^ and microwave pulses and is described in more detail in the Methods section.

### Displacement amplitude measurement

**Fig. 1:**
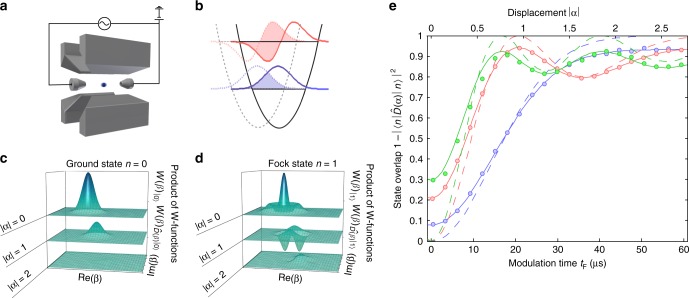
Measuring displacement amplitudes. **a** Schematic of the experimental setup: a magnesium ion is trapped in a linear Paul trap and an additional ac voltage on the end electrodes implements the motional displacement (coherent excitation of motion). **b** Illustration of the wave function overlap. Initial motional state wave functions are shown as dashed lines (*n* = 0 blue, *n* = 1 red). After applying a resonant oscillating force, the ion’s motion is in a displaced Fock state whose wave function is depicted by the solid colored lines. To infer the displacement, the wave function overlap is measured as sketched by the colored areas. **c, d** Illustration of the mechanism behind enhanced sensitivity of Fock states to displacements. **c** and **d** show the theoretically calculated product of the initial and final Wigner function for three different displacements for the Fock states *n* = 1 and *n* = 0, respectively. By integrating over the whole phase space it is possible to infer the state overlap shown in **e**. The negative parts of the *n* = 1 Fock states lead to a vanishing integral for *α* = 1, before the contours of the Wigner functions are fully separated. For states without negative regions in the Wigner function, such as Gaussian states, the integral can only vanish when the product of Wigner functions vanishes over the whole phase space. **e** The graph shows the outcome of the state overlap measurement for three different initial Fock states (blue: *n* = 0, red: *n* = 1, green: *n* = 2). The oscillating force is applied for different duration *t*_F_. The solid curves are fits of the equation given in the main text to the data and the dashed lines are the corresponding theoretical curves assuming full contrast. The fit for the motional ground state is used to calibrate the upper *x*-axis, denoting the displacement amplitude |*α*|. Error bars for the standard error of the mean (s.e.m.) due to quantum projection noise are too small to be seen. Each point is an average of approximately 10000 (*n* = 0) or 3000 (*n* = 1, *n* = 2) experiments

Figure 1a shows the principle and Fig. 1e the result of the displacement amplitude measurement for three different initial Fock states (*n* = 0, 1, and 2). The expected state overlap is given by $\left| {\langle n|D(\alpha )|n\rangle } \right|^2 = {\mathrm{exp}}\left( { - |\alpha |^2} \right)\left( {{\cal{L}}_n\left( {|\alpha |^2} \right)} \right)^2$, with the Laguerre polynomials ${\cal{L}}_n$^21^. The measurement suffers from reduced contrast due to imperfections in state preparation and the detection process, which are of technical nature and pose no fundamental limitation. To account for these imperfections the fitting function depicted by the solid line in Fig. 1c is $P_{{\mathrm{fit}}} = C_1 + C_2\,{\mathrm{exp}}\left( { - |\dot \alpha t_{\mathrm{F}}|^2} \right)\left( {{\cal{L}}_n\left( {|\dot \alpha t_{\mathrm{F}}|^2} \right)} \right)^2,$ with free parameters *C*_1_, *C*_2_, and $\dot \alpha$. The fitted value of $\dot \alpha$ for the *n* = 0 data is used to calibrate the displacement strength shown on the upper *x*-axis. The offset and reduced contrast, described by the parameters *C*_1_ and *C*_1_, respectively, are mainly caused by off-resonant Raman scattering during the detection pulses (see Methods).

In contrast to the monotonous behavior of the *n* = 0 measurement outcome, the data for the excited Fock states exhibit fringes due to interference in phase space^22^. The interference fringes and the resulting metrological gain of Fock states can be intuitively understood as a consequence of the negative regions of the Wigner function as shown in Fig.1d. In phase space the overlap of two quantum states is represented by the integral over the product of the Wigner functions

1 $$\left| {\langle \psi _{\mathrm{i}}|\psi _{\mathrm{f}}\rangle } \right|^2 = {\int\!\!\!\!\!\int} {{\mathrm{d}}\beta W(\beta )_{|\psi _{\mathrm{i}}\rangle }W(\beta )_{|\psi _{\mathrm{f}}\rangle }.}$$

**Fig. 2:**
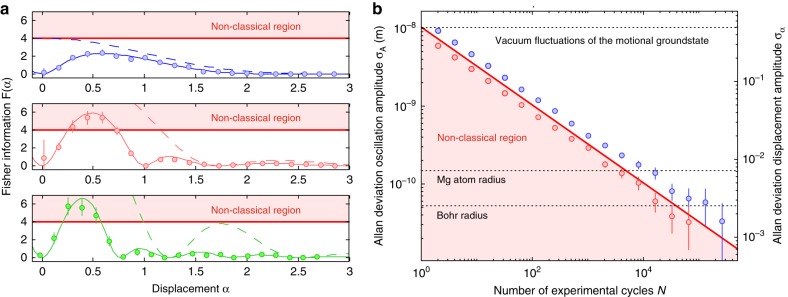
Evaluation of displacement measurement. **a** Fisher information extracted from the amplitude measurement for three different initial Fock states (blue: *n* = 0, red: *n* = 1, green: *n* = 2). The dashed lines show the theoretically expected value for full contrast, whereas the solid lines show the expected values considering the reduced contrast and offset (extracted from fit, see Fig. 1). The additional dips in the Fisher information arise from a vanishing signal slope in the presence of finite technical noise. The standard quantum limit is depicted by the red line. The Fock states with *n* > 0 significantly surpass this limit. The error bars indicate the standard deviation (see Supplementary Note 2 for more details). **b** Allan deviation for an amplitude measurement around *α* = 0.59 with a coherent state (blue circles) and with a *n* = 1 displaced Fock state (red circles). The solid line shows the quantum projection noise limit from Eq. 2 with the classical theoretical optimum ${\cal{F}}_{n = 0} = 4$

In consequence the overlap between a classical state (with positive Wigner function) and its displaced counterpart only vanishes for vanishing overlap of the phase-space contours of the involved states (see Fig. 1c). However, if the quantum state reveals negative values in the Wigner function, as is the case for Fock states, the negative parts in the product can cancel the positive parts and lead to vanishing overlap before the wave packets are spatially separated (see Fig. 1d). The metrological gain is quantified by the Fisher information ${\cal{F}}$ for the displacement measurement, which can be extracted from the data shown in Fig. 1 (see Methods for details). The result is shown in Fig. 2a. For a displacement of *α* = 0.59 the measured Fisher information for the *n* = 1 Fock state measurement is ${\cal{F}}_{n = 1} = 5.37(63)$ (error is standard deviation (s.d)), which implies a metrological gain of $g_{{\mathrm{SQL}}} = \frac{{{\cal{F}}_{n = 1}(\alpha = 0.59)}}{{{\cal{F}}_{{\mathrm{SQL}}}}} = 1.3\,{\mathrm{dB}}$ compared to the theoretical SQL, ${\cal{F}}_{{\mathrm{SQL}}} = 4$, and $g = \frac{{{\cal{F}}_{n = 1}(\alpha = 0.59)}}{{{\mathrm{max}}_\alpha ({\cal{F}}_{n = 0})}} = 3.6\,{\mathrm{dB}}$ compared to the achieved performance for the *n* = 0 state (${\cal{F}}_{n = 0}(\alpha = 0.59) = 2.36(30)$). This corresponds to a reduction in averaging time by more than a factor of two for the same displacement resolution. The Fisher information is directly linked to the achievable measurement uncertainty by the Cramér-Rao bound

2 $${\mathrm{\Delta }}\alpha \ge {\mathrm{\Delta }}\alpha ^{{\mathrm{CR}}} = \frac{1}{{\sqrt {N{\cal{F}}(\alpha )} }},$$

where *N* is the number of independent experimental cycles. In agreement with the Cramér–Rao bound, the uncertainty for the displacement measurement shown in Fig. 2b in the form of an Allan deviation *σ*_*α*_ averages down faster for the n = 1 Fock state (red circles) compared to the ground state (blue circles). The Allan deviation has been calculated from the measured state overlap $|\langle n|D(\alpha )|n\rangle |^2$ and the pre-determined slope of the signal from Fig. 1e. Note that for white noise, the Allan deviation *σ*_*α*_ and standard deviation Δ*α* are identical. The achieved resolution for displacement of $\sigma _A\left( {N = 2^{16}} \right) = 65(23)\,{\mathrm{pm}}$ for *n* = 0 and $\sigma _A\left( {N = 2^{16}} \right) = 32(18)\,{\mathrm{pm}}$ for *n* = 1 can be translated into force measurement resolution (see Supplementary Note 1) of 1.8(0.6) yN for *n* = 0 and 0.9(0.5) yN for *n* = 1 after *N* = 2^16^ = 65536 independent experiments, where an experimental cycle takes 8.1 ms and 9.5 ms for the *n* = 0 and *n* = 1 measurement, respectively.

For displacements generated by $\hat R(\phi _{{\mathrm{LO}}}) = \left( {{\mathrm{sin}}\left( {\phi _{{\mathrm{LO}}}} \right)\hat X \, + {\mathrm{cos}}\left( {\phi _{{\mathrm{LO}}}} \right)\hat P} \right)/\sqrt 2$ with a fixed phase *ϕ*_LO_ the required resource for the quantum enhancement can be identified as non-classicality in terms of the Glauber–Sudarshan P-distribution^23^. Here, however, we consider the more challenging scenario of displacement sensing with an unknown phase. A suitable figure of merit in this case is the sensitivity minimized over all phases. We show in Supplementary Note 4 that this quantity is maximized by pure non-Gaussian states, which necessarily have a negative Wigner function^24^. Furthermore, we show that Fock states are optimal for phase-insensitive displacement sensing. The quantum gain provided by Fock states of *n* > 0 is independent of the phase as their quantum Fisher information *F*_*Q*_ = 8*n* + 4 does not depend on *ϕ*_LO_. It is an interesting open question if phase-insensitive displacement sensing beyond the SQL can in general be linked to negativity of the Wigner function.

### Phase measurement

**Fig. 3:**
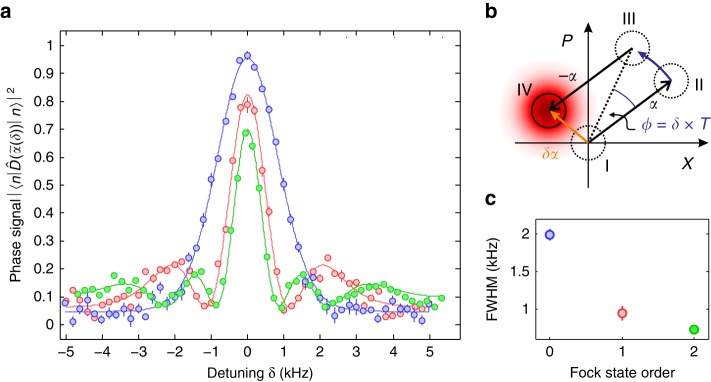
Trapping frequency measurement. **a** Center fringe of the Ramsey pattern for three different initial Fock states (blue: *n* = 0, red: *n* = 1, green: *n* = 2). The circles show the experimental data, which is the population probability of the ion in the $\left| \downarrow \right\rangle$-state ($\left| \uparrow \right\rangle$ for *n* = 0, see text for details). The solid lines show a fit of the theoretically expected lineshape to the data (see Supplementary Note 3). **b** shows a sketch of the phase-space trajectory during the sequence (see text for details). It is assumed that $t_F \ll 1/\delta$, otherwise the phase space trajectories would be curved. **c** shows the full-width-half-maximum (FWHM) extracted from a Gaussian fit to the data. Each data point consists of 1000 experimental cycles, evaluated with a distribution fit technique^19^

As a consequence of the insensitivity of the Fock state to the displacement direction, the same state can be employed for quantum-enhanced sensing of displacement amplitude and phase changes. We demonstrate this feature by measuring the oscillation frequency of the trapped ion with sub-SQL resolution in a Ramsey-like experiment as sketched in Fig. 3b. The Ramsey sequence starts with the initialization of the ion’s motion in a Fock state (I) and a subsequent displacement in phase space (II). If the drive for the displacement was detuned by *δ* from the trap frequency, the displaced state will evolve in phase space on a circle around the origin and accumulate a phase *ϕ* = *δ* × *T* compared to the driving field during the waiting time *T* (III). Undoing the displacement (IV) maps this phase onto a residual displacement $\tilde \alpha$ that can be detected with the overlap detection method introduced above. The center fringe of the Ramsey pattern for waiting time *T* = 50 μs and initial displacement *α* = 1.6 is shown in Fig. 3a. As illustrated by the data shown in Fig. 3c, the width of the center fringe decreases with increasing Fock state order. The full-width-half-maximum (FWHM) is extracted from a Gaussian fit to the center peaks. Note that a narrower width does not necessarily imply a metrological gain. For an increase in Fisher information the slope of the line feature has to increase. For *n* = 2 the reduction in linewidth is fully compensated by the reduced contrast. The whole Ramsey pattern for the different initial Fock states is shown in Supplementary Fig. 1 and the theoretical lineshape is derived in Supplementary Note 3.

**Fig. 4:**
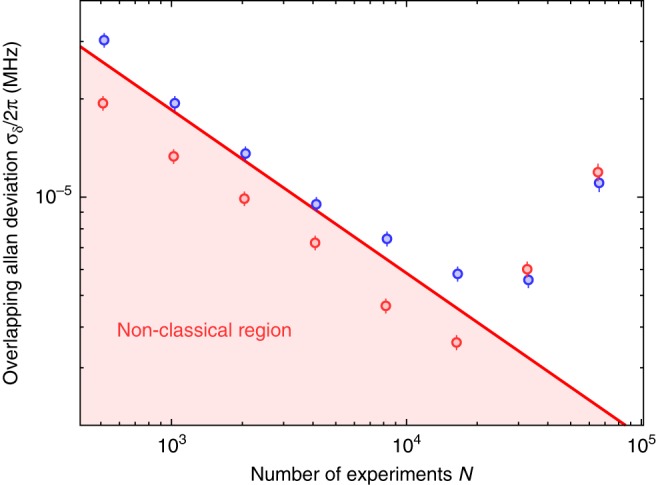
Evaluation of trapping frequency measurement. Overlapping Allan deviation for frequency measurement with a coherent state (blue circles) and with a *n* = 1 displaced Fock state (red circles). The solid line shows the quantum projection noise limit from Eq. 3

To evaluate the performance of the quantum sensing techniques, we have performed a trapping frequency measurement by two-point sampling and analyzed the data in terms of an Allan deviation (see Fig. 4). Since the *n* = 2 Fock state in our case does not provide an additional metrological advantage (see Fig. 2) as a consequence of the reduced contrast caused by technical limitations of the implementation, we have performed the Allan deviation analysis for the *n* = 0 and *n* = 1 Fock state only. The measurement has been performed in an interleaved pattern with an average cycle time of 6.6 ms and 7.8 ms for the *n* = 0 and *n* = 1 measurement, respectively. The Allan deviation for the *n* = 0 protocol averages down to $\sigma _\delta ^{n = 0} = 2\pi \times 5.8(3)\,{\mathrm{Hz}}$. The achievable resolution is limited by a linear drift of the trapping frequency, which leads to an increase in the Allan deviation for long averaging times. The red line in Fig. 4 is the SQL given by

3 $$\sigma _\delta ^{{\mathrm{SQL}}} = \frac{1}{{2\left( {T + t_F} \right)}}\frac{1}{{|\alpha |\sqrt N }},$$

which is the lowest statistical uncertainty achievable with a classical state (Supplementary Note 5). For the quantum-enhanced technique with *n* = 1, the overlapping Allan deviation reaches $\sigma _\delta ^{n = 1} = 2\pi \times 3.6(2)\,{\mathrm{Hz}}$ before it increases due to the linear drift. Using the *n* = 1 Fock state improves the frequency resolution by more than 60% compared to the vacuum state. This is a direct consequence of the quantum-enhanced reduction in averaging time, which allows measuring the trapping frequency with high accuracy before it starts drifting.

## Discussion

In summary, we have demonstrated a quantum-enhanced sensing scheme based on motional Fock states to measure the amplitude and the phase of an oscillating force with resolution below the standard quantum limit. The demonstrated sensing scheme is conceptually different from a previously demonstrated quantum-enhanced method to measure motional frequencies based on phase-sensitive superpositions of Fock states^25^ that has recently been improved^26^. In contrast to the scheme presented here, amplitude measurements are not accessible with this technique. The Fock state sensing scheme does not require any phase relation between the displacement and the quantum state of the detector, which is an important feature when measuring arbitrary interactions without prior phase information (see Supplementary Fig. 2). Previously implemented phase-insensitive schemes exploited correlated modes of atomic ensembles^3,27^, while our scheme requires no mode entanglement.

A technological application of this technique is the measurement of small rf signals applied to a suitable electrode of the ion trap^28^ with enhanced signal-to-noise ratio. Quantum logic spectroscopy^29^ based on motional displacements^30,31^ will benefit from the presented amplitude detection technique, in particular for state detection and spectroscopy of non-closed transitions^32^, where scattering on the spectroscopy ion has to be reduced to a minimum. Specifically, this approach may help to find narrow transitions in highly-charged ions (HCI) that are typically only known with large uncertainty^33^. The small displacement exerted by an optical standing wave tuned near a narrow resonance of a HCI can be detected for larger detunings using the demonstrated Fock state metrology scheme, thus reducing the time to find the transition. Further, these schemes benefit from the phase insensitivity, because the initial motional state is in general produced by manipulating the logic ion with a laser that is independent from the spectroscopy laser. Applications of quantum-enhanced spectroscopy are tests for variation of fundamental constants using molecular ions^34,35^, highly-charged ions^33^, and optical clocks^1,31^. Isotope shift measurements^36,37^ based on photon recoil spectroscopy profit from an improved detection of the small displacement of scattered photons^10,30^ and probe nuclear structure and new physics effects^38–41^.

The presented quantum-enhanced frequency measurement can help to further improve high precision mass measurements of atoms in Paul traps^42^ and g-factor measurements of subatomic particles, such as (anti-)protons in Penning traps^43,44^. Both cases will benefit from a quantum logic approach, in which a mass or spin-dependent force on the particle of interest is probed with quantum-enhanced sensitivity by a nearby well-controllable logic ion using motional Fock states.

In Supplementary Note 6, the analogy to a general two mode interferometer is drawn, which shows that the presented scheme can in principle also be applied to optical and atomic interferometers that have widespread applications from gravitational wave detection^2^ to inertial sensing^45^.

Further improvements in sensitivity can be achieved by employing techniques that allow the generation and overlap detection of larger Fock states with high fidelity. Scalable overlap measurements for Fock states up to *n* = 10 have been reported^46^, allowing phase-insensitive suppression of quantum projection noise of up to 13.2 dB.

## Methods

### Trap modulation to implement displacement operator

Applying a resonantly oscillating electric field at the position of the ion leads to a displacement of the ion’s motional state in phase space^47^. The interaction Hamiltonian for a trapped ion with an additional time-dependent potential $V(t,z) = - qE(t)\hat z$, where *q* and $\hat z$ are the charge and the position of the ion, respectively, and *E*(*t*) is the time-dependent electric field, that is assumed to be spatially constant over the extent of the ion’s wave function, can be written as

4 $$\hat H = - qE(t)z_0\left( {\hat a{\mathrm{e}}^{ - {\mathrm{i}}\omega _zt} + \hat a^\dagger {\mathrm{e}}^{{\mathrm{i}}\omega _zt}} \right),$$

in an interaction picture with respect to the free harmonic oscillation Hamiltonian $\hat H_{{\mathrm{HO}}} = \hbar \omega _z\hat a^\dagger \hat a$ and $\hat z$ is the position operator $\hat z = z_0\left( {\hat a{\mathrm{e}}^{ - {\mathrm{i}}\omega _zt} + \hat a^\dagger {\mathrm{e}}^{{\mathrm{i}}\omega _zt}} \right)$ with the annihilation(creation) operator $\hat a(\hat a^\dagger )$ and ground state wave function extent $z_0 = \sqrt {\hbar /2m\omega _z}$. For an electric field oscillating at the trapping frequency *ω*_*z*_, this leads to the static Hamiltonian

5 $$\hat H = - \frac{{qE_0z_0}}{2}\left( {\hat a{\mathrm{e}}^{ - {\mathrm{i}}\phi _{{\mathrm{LO}}}} + \hat a^\dagger {\mathrm{e}}^{{\mathrm{i}}\phi _{{\mathrm{LO}}}}} \right),$$

where fast oscillating terms (at twice the trapping frequency) are neglected within the rotating wave approximation. Here, *ϕ*_LO_ and *E*_0_ are the phase and amplitude of the driving field, respectively. The unitary evolution according to this Hamiltonian is

6 $$\hat U(t) = {\mathrm{e}}^{ - {\textstyle{i \over h}}\hat Ht} = \hat D(\alpha )$$

and can be identified as the displacement $\hat D(\alpha ) = {\mathrm{e}}^{\alpha \hat a^\dagger - \alpha ^ \ast \hat a}$ operator with displacement amplitude $\alpha = \frac{{{\mathrm{i}}qE_0z_o}}{{2\hbar }}{\mathrm{e}}^{{\mathrm{i}}\phi _{{\mathrm{LO}}}} \times t$.

### Overlap measurement

**Fig. 5:**
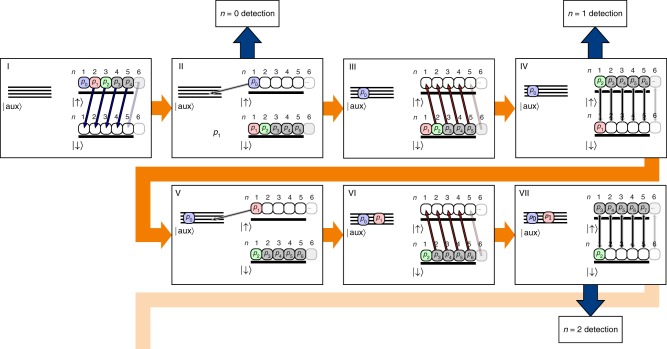
Experimental scheme to measure the motional state overlap with Fock state *n*. The reduced level scheme shows the spin states |↓〉, |↑〉 and the manifold of auxiliary states |aux〉 (see text for details). The boxes indicate the current motional state within the sequence. The values *p*_*n*_ denote the initial population of the Fock state *n*. The sequence starts with the ion in the |↑〉 state and an unknown motional state distribution.(I) A blue sideband RAP pulse flips the spin and removes a phonon from all excited Fock states, leaving the ground state population untouched. (II) The motional ground state population is measured via spin state-selective fluorescence. To detect the population of higher order Fock states, the ground state population is hidden by a sequence of rf-pulses in one of the auxiliary states |aux〉_1_. (III) A red sideband RAP pulse flips the spin and removes a phonon from all excited Fock states |↓〉. Therefore only the population that was initially in the *n* = 1 Fock state remains in the bright spin state. (IV) Detection of the ions spin gives the *n* = 1 Fock state population. Alternatively, step (II)–(IV) can be repeated after a rf carrier *π*-flop to detect higher order Fock states (see (V)–(VII)). However, only a single selected Fock state population can be measured in a single experiment

All measurements described in the manuscript rely on the ability to measure the motional state population in a given Fock state. To achieve this, we have implemented a sequence that transfers a selected initial population *p*_*n*_ to the motional and electronic ground state, while all other motional population is in the |↑〉 state. State-selective fluorescence then provides the population *p*_*n*_. The sequence for measuring *p*_0_, *p*_1_ and *p*_2_ is shown in Fig. 5. The ion is initialized in the |↑〉-state. At the beginning of the detection sequence the motional population {*p*_*n*_} is distributed over several motional Fock states *n*. (I). A blue sideband rapid adiabatic passage pulse (RAP) transfers the internal state to |↓〉, while simultaneously taking out a quantum of motion, therefore keeping the ground state population untouched^20^. Averaging the number of |↓〉 and |↑〉 detection events after this mapping step provides the *n* = 0 population. For higher order Fock state detection the protocol has to be extended as follows. The ground state population can be hidden in a dark auxiliary state |aux〉 by radio frequency pulses (II). In ^25^Mg^+^ the Zeeman substates with *m*_*F*_ = 1, 0, −1, −2 of the *F* = 2 dark hyperfine state can be used for this purpose. A second sideband RAP pulse (III), this time on the red sideband, flips the spin for all motional states except for the ground state, which stores the information about the initial Fock state *n* = 1 population. Fluorescence detection of the ion’s spin will give the initial *n* = 1 Fock state population. To detect even higher Fock states, the spin is flipped independent of the motional state to initialize the |↑〉-state again (IV). Now steps (II)–(IV) are repeated until the desired Fock state population is isolated in the state |↓〉 from the rest of motional population (e.g., see (V)–(VII) for *n* = 2). Reduced contrast due to off-resonant scattering during the involved RAP pulses is the main limitation in our experiments. We estimate single *π*-flop fidelities on sideband transitions to be above 95%. However, the detection sequence in a protocol for Fock state *n* requires *n* + 1 RAP pulses with a pulse area of around 10 *π*-times resulting in a loss of contrast of around 10% per RAP pulse. This limitation can be overcome by operating the Raman laser with a larger detuning, which requires higher laser power, or ion species providing an optical qubit such as Ca^+^ that do not suffer from this limitation. The ultimate limitation for high *n* is the limited number of auxiliary states available in ^25^Mg^+^. However, other techniques for phonon counting up to *n* = 10 by exploiting trap induced Kerr-nonlinearities have been demonstrated^46^ and modifications using laser-induced Kerr-nonlinearities^48^ combined with continuous dynamic decoupling techniques^49^ might be an option for future implementations.

### Quantum metrology

The precision of an estimation is bounded by means of the Cramér–Rao bound as

7 $${\mathrm{\Delta }}\theta _{{\mathrm{est}}} \ge {\mathrm{\Delta }}\theta _{{\mathrm{CR}}} = \frac{1}{{\sqrt {N{\cal{F}}(\theta )} }},$$

where *θ*_est_ is an arbitrary estimator for *θ*, *N* is the number of repeated measurements, and

8 $${\cal{F}}(\theta ) = \mathop {\sum}\limits_\mu {\frac{1}{{P(\mu |\theta )}}\left( {\frac{{\partial P(\mu |\theta )}}{{\partial \theta }}} \right)^2}$$

is the (classical) Fisher information. The probability distribution $P(\mu |\theta ) = {\mathrm{Tr}}\{ {\hat{\mathrm{{\Pi}}}}_\mu \hat \rho (\theta )\}$ is determined by the quantum state $\hat \rho (\theta )$ and the choice of measurement, described by the projectors $\{ {\hat{\mathrm{{\Pi}}}}_\mu \} _\mu$. We consider scenarios in which the unknown phase *θ* is imprinted by a unitary process, i.e. $\hat \rho (\theta ) = \hat U(\theta )\hat \rho \hat U(\theta )^\dagger$ with $\hat U(\theta ) = e^{ - i\hat H\theta }$.

The mean value $\langle \hat M\rangle _{\hat \rho (\theta )} = {\mathrm{Tr}}\{ \hat M\hat \rho (\theta )\}$ and variance $\left( {{\mathrm{\Delta }}\hat M} \right)_{\hat \rho (\theta )}^2 = \left\langle {\hat M^2} \right\rangle _{\hat \rho (\theta )} - \left\langle {\hat M} \right\rangle _{\hat \rho (\theta )}^2$ of the measured observable $\hat M = \mathop {\sum}\limits_\mu {\mu {\hat{\mathrm{{\Pi}}}}_\mu }$ can be used to derive a lower bound for the Fisher information^50^

9 $${\cal{F}}(\theta ) \ge \frac{1}{{({\mathrm{\Delta }}\hat M)_{\hat \rho (\theta )}^2}}\left( {\frac{{d\langle \hat M\rangle _{\hat \rho (\theta )}}}{{d\theta }}} \right)^2.$$

This bound is tight if there are only the two measurement outcomes *μ* = 1, 0 with $P(1|\theta ) = 1 - P(0|\theta )$ and $({\mathrm{\Delta }}\hat M)_{\hat \rho (\theta )}^2 = P(1|\theta )(1 - P(0|\theta ))$.

Maximizing the Fisher information over all possible measurements leads to the quantum Fisher information^51^

10 $$\begin{array}{*{20}{c}} {{\mathrm{max}}} \\ {\{ {\hat{\mathrm{{\Pi}}}}_\mu \} } \end{array}{\cal{F}}(\theta ) = {\cal{F}}_Q[\hat \rho ,\hat H],$$

which is a function of the initial state $\hat \rho$ and the generator $\hat H$ of the unitary evolution. We obtain the quantum Cramér–Rao bound as the general precision limit for quantum parameter estimation^52^

11 $${\mathrm{\Delta }}\theta _{{\mathrm{est}}} \ge {\mathrm{\Delta }}\theta _{{\mathrm{CR}}} \ge {\mathrm{\Delta }}\theta _{{\mathrm{QCR}}} = \frac{1}{{\sqrt {N{\cal{F}}_Q[\hat \rho ,\hat H]} }}.$$

### Extracting the Fisher information from experimental data

We can use the data shown in Fig. 1c to get a measured value for the Fisher information of our measurement. As can be seen from Eq. 9, the Fisher information depends on the slope and the noise properties of the measurement presented before. The slope $s(\alpha _i) = \frac{{d\langle \hat M\rangle _{\hat \rho (\alpha )}}}{{d\alpha }}$ is experimentally determined for each displacement amplitude *α*_*i*_ by a symmetric difference quotient

12 $$s(\alpha _i) = \frac{{P_{| \downarrow \rangle }(\alpha _{i + 1}) - P_{| \downarrow \rangle }(\alpha _{i - 1})}}{{\alpha _{i + 1} - \alpha _{i - 1}}}.$$

For the first and last measurement point is determined by an asymmetric difference quotient

13 $$s(\alpha _i) = \frac{{P_{| \downarrow \rangle }(\alpha _{i + 1}) - P_{| \downarrow \rangle }(\alpha _i)}}{{\alpha _{i + 1} - \alpha _i}}$$

As discussed before, the noise is dominated by quantum projection noise.

### Oscillation amplitude

For a harmonic oscillator, the position observable $\hat x$ is related to the quadrature component $\hat X = \frac{1}{{\sqrt 2 }}\left( {\hat a^\dagger + \hat a} \right)$ by

14 $$\hat z = \sqrt {\frac{\hbar }{{m\omega _z}}} \hat X$$

From this relation the expectation value of the position operator for a coherent state $\alpha$ can be evaluated to be

15 $$\langle \hat z\rangle _\alpha = \sqrt {\frac{\hbar }{{2m\omega _z}}} 2\alpha \,{\mathrm{cos}}\,\omega _zt = 2z_0\alpha \,{\mathrm{cos}}\,\omega _zt.$$

Therefore the oscillation amplitude for a given displacement is *A* = 2*z*_0_*α*. Accordingly, the *y*-axis in Fig. 2b was scaled by Δ*A* = 2*z*_0_Δ*α*.

## Supplementary information

Supplementary Information

Description of Additional Supplementary Files

Supplementary Movie 1

Supplementary Movie 2

Supplementary Movie 3

Source Data

## Data Availability

All data that support the findings presented in this manuscript are available from the corresponding author upon reasonable request. The source data underlying Figs 1c, 2a,b, 3a, c and 4 are provided as a Source Data file.
