# Supplementary material for: Motional Fock states for quantum-enhanced amplitude and phase measurements with trapped ions
Source: Nat Commun. 2019 Jul 2;10:2929. doi: 10.1038/s41467-019-10576-4 (PMC6606596; doi:10.1038/s41467-019-10576-4)
Supplement: Supplementary file 1 — Supplementary Information [file 41467_2019_10576_MOESM1_ESM.pdf]

Supplementary Material for

Motional Fock states for quantum-enhanced amplitude and phase measurements with trapped ions

Wolf et al.

Supplementary Note 1 Estimation of the achievable force sensitivity

The amplitude F of a resonant oscillating force required to get a displacement of α after time t_{F} is given by

$$F = \frac{2\hbar}{z_0 t_{\mathrm{F}}} \times \alpha, \tag{1}$$

where $z_0 = \sqrt{\hbar/2m\omega_z}$ is the ground state wave packet extent for an atom with mass m, trapped in a harmonic potential with trap frequency ω_z. Therefore, the statistical uncertainty for a force estimation can be written as

$$\begin{aligned} \Delta F &= \frac{2\hbar}{z_0 t_{\mathrm{F}}} \times \Delta\alpha & (2)\\ &\geq \frac{2\hbar}{z_0 t_{\mathrm{F}}} \frac{1}{\sqrt{\mathcal{F}N}}, & (3) \end{aligned}$$

with $\mathcal{F}$, the Fisher information for the α-estimation (measured result shown in Fig. 2 a) and N the number of experiments. Rewriting this expression with $N = \tau/t_{\mathrm{cycle}}$, and introducing $R_F = t_{\mathrm{cycle}}/t_{\mathrm{F}}$ as the ratio of cycle time and t_{F} we obtain

$$\frac{\Delta F}{\sqrt{\Delta_c}} \geq \frac{2\hbar R_F}{z_0} \sqrt{\frac{1}{\mathcal{F}\tau}}, \tag{4}$$

where $\Delta_c = 1/t_{\mathrm{cycle}}$ is the measurement bandwidth and τ the total measurement time. For $^{25}\mathrm{Mg}^+$ with $\omega_z = 2\pi \times 1.89\,\mathrm{MHz}$, $t_{\mathrm{F}} = 10\,\mu\mathrm{s}$, $t_{\mathrm{cycle}} = 15\,\mathrm{ms}$, $\mathcal{F} = 5$ and $\tau = t_{\mathrm{cycle}}$ the resulting force sensitivity is $112\,\mathrm{yN}/\sqrt{\mathrm{Hz}}$. It can be enhanced by one to two orders of magnitude by applying a smaller force for a longer time (see Eq. 2). For comparison, Biercuk *et al.* achieved a sensitivity of around $390\,\mathrm{yN}/\sqrt{\mathrm{Hz}}$ [1] with 100 ions. Shaniv and Ozeri demonstrated a sensitivity of $28\,\mathrm{zN}/\sqrt{\mathrm{Hz}}$ [2]. Both schemes rely on Doppler velocimetry. In contrast to that, the scheme presented here relies on a projective measurement, which is ideally capable of saturating the quantum Fisher information.

In a more general framework that is used in the context of heating rate measurements in ion traps, fluctuating electric fields exert forces onto the trapped ions [3]. The electric field is characterized by the single-sided electric field spectral density

$$S_E(\omega) = 2\int_{-\infty}^{\infty} \mathrm{d}\tau \langle E(\tau)E(0)\rangle \mathrm{e}^{-\mathrm{i}\omega\tau}. \tag{5}$$

According to [4] the electric field spectral density can be related to the rate of motional excitation from the motional ground state to the first excited motional Fock state Γ by

$$\Gamma \approx \frac{q^2}{4m\hbar\omega_z} S_E(\omega_z), \tag{6}$$

where q is the charge of the ion. It can be seen, that the change in motional excitation only depends on the electric field spectral density at the trapping frequency ω_z. The root-mean-square force F_{rms} can be inferred from the electric field spectral density by

$$F_{\mathrm{rms}} = \frac{q}{\sqrt{2\pi}} \sqrt{\int_0^{\infty} \mathrm{d}\omega S_E(\omega)}. \tag{7}$$

Consequently, in general it is not possible to infer a force from a measurement of the motional excitation only, but additional knowledge about the spectrum of the oscillating field is required.

The scenario treated in the manuscript corresponds to an electric field spectral density that is narrower than the Fourier-limited bandwith of the measurement. Therefore, we can assume a Fourier-limited, nearly monochromatic oscillating electric field $E(t) = E_0 \cos(\omega t)$ applied for a time t_{F} with

$$S_E^{\mathrm{coh}}(\omega) = \frac{1}{2} E_0^2 t_{\mathrm{F}} \left[\mathrm{sinc}\left(\frac{1}{2} t_{\mathrm{F}}(\omega - \omega_0)\right)^2 + \mathrm{sinc}\left(\frac{1}{2} t_{\mathrm{F}}(\omega + \omega_0)\right)^2 \right].$$

Inserting the electric field spectral density into Supplementary Eq. 6, we find

$$\Gamma = \frac{z_0^2 F^2}{4\hbar^2} t_{\mathrm{F}} \tag{8}$$

For a coherent drive, as considered in the manuscript, we can use $|\langle 0|\alpha\rangle|^2 = \mathrm{e}^{-|\alpha|^2} \approx 1 - |\alpha|^2$ assuming that the displacement is small. Therefore the rate of transfering population from motional Fock state $|0\rangle$ to $|1\rangle$ is given by $\Gamma t_{\mathrm{F}} = |\alpha|^2$ which gives us Supplementary Eq. 1.

This formalism can also be used to derive a lower bound on detectable forces in the presence of heating with a rate $\dot{n}$, which was done before by Maiwald et al. [5]. To distinguish a motional excitation $\langle n_{\mathrm{coh}}\rangle = |\alpha|^2$ due to a force signal from unintended motional heating $\langle n_{\mathrm{h}}\rangle = \dot{n} t_{\mathrm{F}}$, the inequality $\langle n_{\mathrm{coh}}\rangle \geq \langle n_{\mathrm{h}}\rangle$ must hold and therefore

$$F \geq \frac{2\hbar}{z_0\sqrt{t_{\mathrm{F}}}}\sqrt{\dot{n}}. \tag{9}$$

It should be noted, that this estimation does not consider the fact that stochastic forces such as motional heating and a coherent drive lead to different motionally excited states, which requires a more detailed treatment that also considers the particular measurement scheme.

Stochastic forces with an electric field spectral density that is much broader than the Fourier-limited bandwidth are another extreme case usually investigated in the context of heating rate measurements. Modelling the electric field spectral density responsible for anomalous heating in ion traps is still subject of investigations [3]. For an order-of-magnitude estimate, we can assume a box-like spectrum centered around ω_z with width Δ_h, i.e.

$$S_E^{\mathrm{h}} = \begin{cases} S_E^{\mathrm{h}}(\omega_z) & \text{if } \omega_z - \frac{\Delta_h}{2} < \omega < \omega_z + \frac{\Delta_h}{2} \\ 0 & \text{else} \end{cases} \tag{10}$$

Under this assumption we can relate a root-mean-square force F_{rms} to the measured heating rate with

$$F_{\mathrm{rms}}^{\mathrm{h}} = q\sqrt{\frac{S_E^{\mathrm{h}}(\omega_z)\Delta_h}{2\pi}} \tag{11}$$

Considering the lowest reported electric field spectral densities from heating rate measurements in the $S_E^{\mathrm{h}} \sim 10^{-15} \frac{\mathrm{V}^2}{\mathrm{m}^2\mathrm{Hz}}$ range [6, 7] we associate a force of $F_{\mathrm{rms}}^{\mathrm{h}} \approx 2\sqrt{\Delta_h} \times 10^{-27} \frac{\mathrm{N}}{\sqrt{\mathrm{Hz}}}$. Note that Δ_h is not the Fourier-limited bandwidth Δ_c in this case but the intrinsic spectral width of the electric field noise that causes the heating, which certainly can exceed the MHz regime.

Supplementary Note 2 Error analysis for Fisher information measurement

Below we will use the shortened notation $P_{|\downarrow\rangle}(\alpha_i) \equiv P_i$ and $s(\alpha_i) \equiv s_i$.

Errors on slope determination

Quantum projection noise The slope is determined by measuring the population at two neighboring points. The error associated with this measurement is $\Delta P_{i-1} = \sqrt{\frac{P_{i-1}(1-P_{i-1})}{N}}$ and $\Delta P_{i+1} = \sqrt{\frac{P_{i-1}(1-P_{i-1})}{N}}$, where N is the number of independent measurements and is propagated to the slope error due to quantum projection noise via

$$(\Delta s_i)_{QPN} = \sqrt{\left(\frac{\partial s_i}{\partial P_{i-1}}\Delta P_{i-1}\right)^2 + \left(\frac{\partial s_i}{\partial P_{i+1}}\Delta P_{i+1}\right)^2} \tag{12}$$

$$= \sqrt{\frac{1}{N}\frac{P_{i-1}(1-P_{i-1}) + P_{i+1}(1-P_{i+1})}{(\alpha_{i+1}-\alpha_{i-1})^2}} \tag{13}$$

Fit error for displacement determination To determine the implemented displacement, we fitted the expected theoretical curve to the measured data for the motional ground state. The fit error $\Delta\alpha$ also gives an error on the denominator in the difference quotient. The resulting slope error reads

$$(\Delta s_i)_{fit} = \frac{s_i}{\alpha_{i+1}-\alpha_{i-1}}\Delta\alpha \tag{14}$$

Error due to finite step size The difference quotient only gives an approximation of the true slope of the signal. For a symmetric derivative the first order error to this approximation is given by

$$(\Delta s_i)_{fin} = \left(\frac{\mathrm{d}^3 P}{\mathrm{d}\alpha^3}\right)_i \frac{(\alpha_{i+1}-\alpha_{i-1})^2}{6}, \tag{15}$$

and in the asymmetric case by

$$(\Delta s_i)_{fin} = \left(\frac{\mathrm{d}^2 P}{\mathrm{d}\alpha^2}\right)_i \frac{(\alpha_{i+1}-\alpha_{i-1})}{2}, \tag{16}$$

For the error estimation we used the theoretically calculated derivative of $P = \exp(-|\alpha|^2)\mathcal{L}_n(|\alpha|^2)^2$, where n denotes the number of excitations in the Fock state, without additional parameters accounting for the reduced contrast observed in the experiment.

Total slope error The total error on the estimation of the signal slope s then reads

$$(\Delta s_i) = \sqrt{(\Delta s_i)^2_{QPN} + (\Delta s_i)^2_{fit} + (\Delta s_i)^2_{fin}} \tag{17}$$

Errors on QPN determination

Since we use a binary data set to determine the expectation value for the spin measurement, the value for the variance is exact. However, for finite N statistical fluctuation will give rise to an uncertainty given by $\Delta QPN_i = P_i(1-P_i)\sqrt{\frac{2}{N-1}}$ [8].

$(\Delta\mathcal{F}_5)_{s_{\mathrm{QPN}}}$	0.427
$(\Delta\mathcal{F}_5)_{\mathrm{fit}}$	0.006
$(\Delta\mathcal{F}_5)_{\mathrm{fin}}$	0.464
$(\Delta\mathcal{F}_5)_{\mathrm{QPN}}$	0.068

Supplementary Table 1: error budget for the Fisher information estimation of measurement point $i = 5$ with $\alpha = 0.59$.

Total error

The total estimated error on the measurement of the Fisher information is given by

$$\Delta\mathcal{F} = \sqrt{\left(\frac{2s}{P_i(1-P_i)}\Delta s_i\right)^2 + \left(\frac{s^2}{(P_i(1-P_i))^2}\Delta QPN_i\right)^2}\,. \tag{18}$$

The most significant violation of the standard quantum limit has been observed for point $i = 5$ in the Fock state $n = 1$ data. The Fisher Information for this measurement was $\mathcal{F}_5 = 5.37(63)$ and the different uncertainties are summarized in the error budget in Supplementary Table 1

Supplementary Note 3 Ramsey pattern line shape

The individual displacement pulses start at t_0 and are applied for a duration t_F. The oscillating force is detuned by δ from the axial trap frequency ω_z, resulting in the time dependent interaction Hamiltonian

$$\hat{H} = i\hbar\left(\gamma(t)\hat{a}^\dagger - \gamma^*(t)\hat{a}\right) \tag{19}$$

where $\gamma(t) = i\Omega e^{i(\delta\cdot t+\phi_{LO})}$ and $\Omega = -qE_0z_0/(2\hbar)$ (see also Methods' section *Trap modulation to implement displacement operator*). The dynamics is given by the unitary evolution [9]

$$\hat{U}(t_0,t) = \hat{D}\big(\alpha(t_0,t)\big)e^{i\Phi(t_0,t)} \tag{20}$$

where the interaction starts at t_0 and has duration t. The displacement and phase are:

$$\alpha(t_0,t) = \int_{t_0}^{t_0+t} \mathrm{d}\tau\,\gamma(\tau) \tag{21}$$

$$\Phi(t_0,t) = \mathrm{Im}\left[\int_{t_0}^{t_0+t}\mathrm{d}\tau\,\gamma(\tau)\int_{t_0}^{\tau}\mathrm{d}\tau'\,\gamma^*(\tau')\right]\,. \tag{22}$$

For the total sequence the evolution is then

$$\hat{U}_\mathrm{tot} = \hat{U}_{\phi_{LO}+\pi}\big(t_0+t_\mathrm{F}+T,t_\mathrm{F}\big)\,\hat{U}_{\phi_{LO}}\big(t_0,t_\mathrm{F}\big) \tag{23}$$

where subscripts represent a phase change of the local oscillator to undo the initial displacement. Up to a global phase this results in a displacement

$$\hat{U}_\mathrm{tot} = \hat{D}\left(\delta\alpha\right) \tag{24}$$

where $\tilde{\alpha} = \alpha(t_0,t_\mathrm{F}) - \alpha(t_0+t_\mathrm{F}+T,t_\mathrm{F})$ with the two contributions

$$\alpha(t_0,t_\mathrm{F}) = \frac{\Omega e^{i\phi_{LO}}}{\delta}\left(e^{i\delta(t_0+t_\mathrm{F})} - e^{i\delta t_0}\right) = \frac{i\Omega t_\mathrm{F}e^{i(\phi_{LO}+\delta t_0)}}{(\delta t_\mathrm{F}/2)}\sin\left(\frac{\delta t_\mathrm{F}}{2}\right)e^{i\delta t_\mathrm{F}/2} \tag{25}$$

$$\alpha(t_0+t_\mathrm{F}+T,t_\mathrm{F}) = \frac{\Omega e^{i\phi_{LO}}}{\delta}\left(e^{i\delta(t_0+2t_\mathrm{F}+T)} - e^{i\delta(t_0+t_\mathrm{F}+T)}\right) = \frac{i\Omega t_\mathrm{F}e^{i(\phi_{LO}+\delta t_0)}}{(\delta t_\mathrm{F}/2)}\sin\left(\frac{\delta t_\mathrm{F}}{2}\right)e^{i\delta(3t_\mathrm{F}+2T)/2}\,. \tag{26}$$

So the residual displacement at the end of the sequence is

$$\tilde{\alpha} = 2\Omega t_\mathrm{F}\,\mathrm{sinc}\left(\frac{\delta\,t_\mathrm{F}}{2}\right)\sin\left(\frac{\delta(T+t_\mathrm{F})}{2}\right)\cdot e^{i\phi_{LO}}e^{i\delta(t_0+t_\mathrm{F}+T/2)} \tag{27}$$

This residual displacement can be detected by the overlap measurement described in the main text and gives the final result for the line shape of the Ramsey pattern

$$|\langle n|\hat{D}(\tilde{\alpha})|n\rangle|^2 = \exp(-|\tilde{\alpha}|^2)\big(\mathcal{L}_n(|\tilde{\alpha}|^2)\big)^2\,. \tag{28}$$

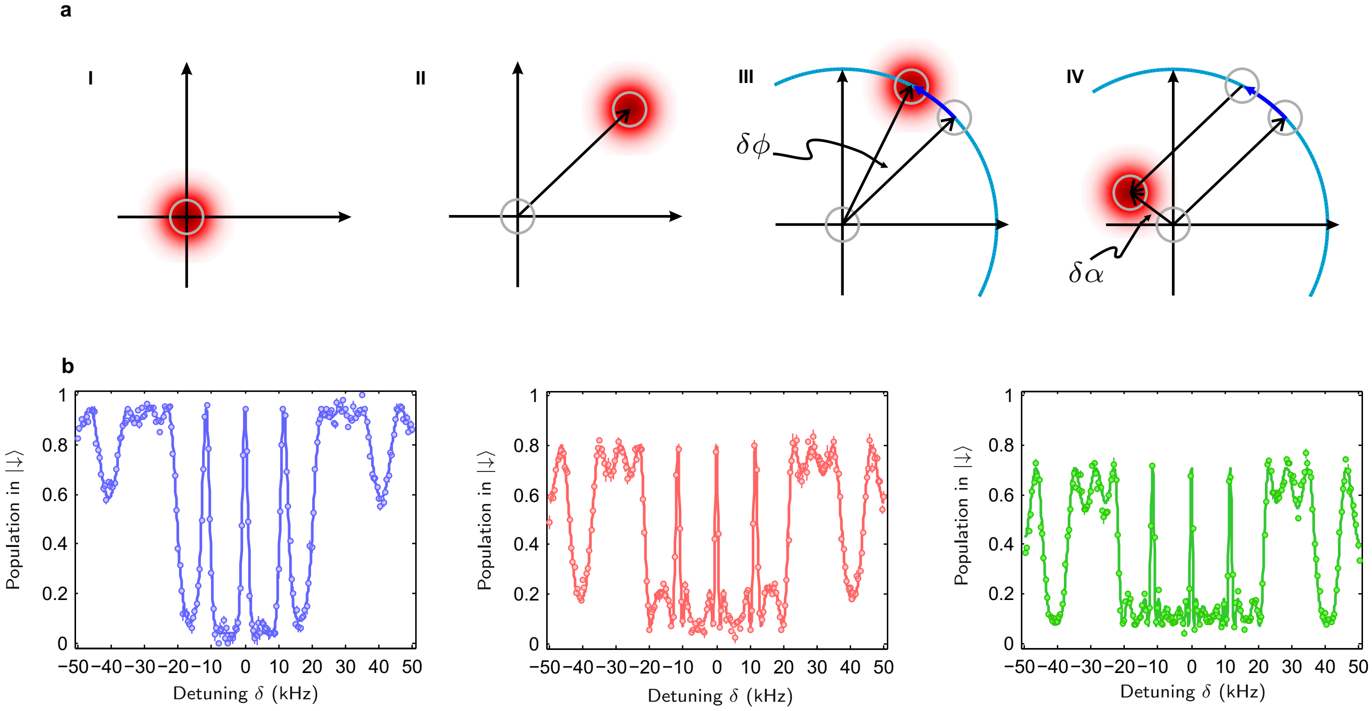

Supplementary Figure 1: **Ramsey scheme for trapping frequency measurement a** Ramsey scheme to measure the trap frequency. (**I**) The ion is prepared in the motional ground state (or higher Fock state, not shown in Figure). After a displacement implemented with a detuned oscillating force (**II**) the ion's motion accumulates a phase $\phi = \delta \times T$ during a waiting time T (**III**). When the displacement is undone (**IV**), a residual displacement remains, which depends on the accumulated phase. Measuring the residual displacement with the overlap measurement technique described above for different detuning gives the Ramsey fringes shown in subfigure **b**. The experimental results are shown for three different Fock states (blue: $n = 0$, red: $n = 1$, green: $n = 2$). It can be seen, that with increasing Fock state order, the width of the resonance lines decreases. Each point is an average of 1250 experiments. The solid line is a fit to the theoretical lineshape (Supplementary Eq. (28)) considering the reduced contrast.

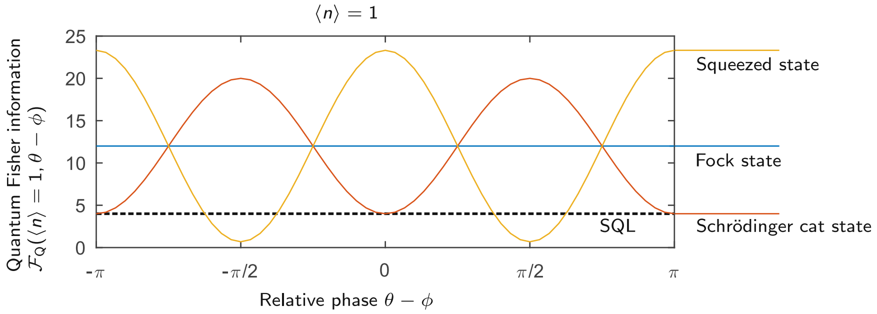

Supplementary Figure 2: The graphic shows the quantum Fisher information for a squeezed state (yellow line), a Schrödinger cat state (red line), and a Fock state (blue line) with $\langle n\rangle = 1$ as a function of the phase between displacement and quantum state. The dashed line indicates the standard quantum limit. Another value for $\langle n\rangle$ rescales the y-axis. It can be seen that the average value for the Fisher information only depends on the mean phonon excitation for states with $\langle\hat{X}\rangle = 0$ and $\langle\hat{P}\rangle = 0$. In the case of displacement sensing with an unknown phase, a suitable figure of merit is the sensitivity minimized over all phases. From the graph, it can be seen that Fock states ($\min_\phi \mathcal{F}_Q(\langle n\rangle = 1) = 12$) outperform Schrödinger cat states ($\min_\phi \mathcal{F}_Q(\langle n\rangle = 1) = 4$) and squeezed states ($\min_\phi \mathcal{F}_Q(\langle n\rangle = 1) \approx 0.69$).

Supplementary Note 4 Optimal estimation of a displacement amplitude without phase information

We consider the metrological task of estimating the amplitude of a displacement. The phase of the displacement is unknown at the time of the state preparation. To optimize the sensitivity of the estimation, the 'detector' shall be prepared in an optimal quantum state.

The unitary process which generates the phase shift is given by the displacement

$$\begin{aligned}\hat{D}(\alpha) &= \exp\left(\alpha\hat{a}^\dagger - \alpha^*\hat{a}\right)\\ &= \exp\left((\hat{a}^\dagger e^{-i\phi_{\text{LO}}} - \hat{a}e^{i\phi_{\text{LO}}})\frac{\theta}{2}\right)\\ &= \exp\left(-i\hat{R}(\phi_{\text{LO}})\theta\right), \end{aligned} \tag{29}$$

where we defined real parameters θ and ϕ_{LO}, such that $\alpha = \theta e^{-i\phi_{\text{LO}}}/2$ and $\hat{R}(\phi_{\text{LO}}) = (\sin(\phi_{\text{LO}})\hat{X} + \cos(\phi_{\text{LO}})\hat{P})/\sqrt{2}$. Furthermore, we used $\hat{X} = (\hat{a} + \hat{a}^\dagger)/\sqrt{2}$ and $\hat{P} = i(\hat{a}^\dagger - \hat{a})/\sqrt{2}$. Our goal is to estimate the parameter $\theta = 2|\alpha|$. To distinguish the Fisher Information with respect to $|\alpha|$, $\mathcal{F}$, from the Fisher information with respect to θ, the latter is denoted by a Gothic type $\mathfrak{F}$. The connection between them is

$$\mathcal{F} = 4\mathfrak{F}\,. \tag{30}$$

The reason for introducing $\mathfrak{F}$ is to normalize the SQL to one, which is a widely used convention in the literature [10], whereas in the main manuscript the classical limit is at 4, but the estimated parameter is $|\alpha|$ from the standard definition of the displacment operator. According to Eq. (7) of the main text, the sensitivity of the estimation of θ is bounded by the quantum Fisher information $\mathfrak{F}_Q[\hat{\rho}, \hat{R}(\phi_{\text{LO}})]$, which depends on the phase ϕ_{LO} via the generator $\hat{R}(\phi_{\text{LO}})$. In a "worst-case" scenario the sensitivity may be reduced to

$$\mathfrak{F}_{\min}[\hat{\rho}] = \min_{\phi_{\text{LO}}} \mathfrak{F}_Q[\hat{\rho}, \hat{R}(\phi_{\text{LO}})]. \tag{31}$$

In order to prepare the detector such as to render it most sensitive, even in this worst-case scenario, we need to maximize the figure of merit $\mathfrak{F}_{\min}[\hat{\rho}]$. An alternative strategy consists in optimizing the average performance, as quantified by the figure of merit

$$\mathfrak{F}_{\text{mean}}[\hat{\rho}] = \frac{1}{2\pi}\int_0^{2\pi} d\phi \mathfrak{F}_Q[\hat{\rho}, \hat{R}(\phi_{\text{LO}})]. \tag{32}$$

Below, we derive the limits on these two figures of merit as a function of the number of excitations. We will see that a Fock state maximizes the sensitivity in both cases.

Optimizing the minimum sensitivity

Let us first focus on the quantity given by Supplementary Eq. (31). Using $\mathfrak{F}_Q[\hat{\rho}, \hat{R}(\phi_{\text{LO}})] \leq 4(\Delta\hat{R}(\phi_{\text{LO}}))^2_{\hat{\rho}}$ [11], we find the following upper bound

$$\begin{aligned}\mathfrak{F}_{\min}[\hat{\rho}] &\leq 4\min_{\phi_{\text{LO}}}(\Delta\hat{R}(\phi_{\text{LO}}))^2_{\hat{\rho}} \\ &= 2\min_{\mathbf{n}} \mathbf{n}^T\Gamma_{\hat{\rho}}\mathbf{n},\end{aligned} \tag{33}$$

where we introduced the 2×2 covariance matrix

$$\Gamma_{\hat{\rho}} = \begin{pmatrix} (\Delta\hat{X})^2_{\hat{\rho}} & \text{Cov}(\hat{X},\hat{P})_{\hat{\rho}} \\ \text{Cov}(\hat{X},\hat{P})_{\hat{\rho}} & (\Delta\hat{P})^2_{\hat{\rho}} \end{pmatrix}, \tag{34}$$

with

$$\text{Cov}(\hat{X},\hat{P})_{\hat{\rho}} = \frac{1}{2}\text{Tr}\{\hat{\rho}(\hat{X}\hat{P}+\hat{P}\hat{X})\} - \text{Tr}\{\hat{\rho}\hat{X}\}\text{Tr}\{\hat{\rho}\hat{P}\}, \tag{35}$$

and a unit vector $\mathbf{n} = (\sin(\phi_{\text{LO}}), \cos(\phi_{\text{LO}}))$. The minimum in Supplementary Eq. (33) is given by the smallest eigenvalue $\lambda_{\min}$ of the matrix $\Gamma_{\hat{\rho}}$. This eigenvalue can again be bounded from above:

$$\begin{aligned}\lambda_{\min}(\hat{\rho}) &= \frac{(\Delta\hat{X})^2_{\hat{\rho}} + (\Delta\hat{P})^2_{\hat{\rho}}}{2} \\ &\quad - \frac{1}{2}\sqrt{((\Delta\hat{X})^2_{\hat{\rho}} - (\Delta\hat{P})^2_{\hat{\rho}})^2 + 4\text{Cov}(\hat{X},\hat{P})^2_{\hat{\rho}}} \\ &\leq \frac{1}{2}\text{Tr}\{\hat{\rho}(\hat{X}^2+\hat{P}^2)\} \\ &= \text{Tr}\{\hat{\rho}(\hat{a}^\dagger\hat{a}+1)\} \\ &= n + \frac{1}{2},\end{aligned} \tag{36}$$

where $n = \text{Tr}\{\hat{\rho}\hat{a}^\dagger\hat{a}\}$ determines the number of excitations. Hence, the minimal sensitivity is generally bounded by

$$\mathfrak{F}_{\min}[\hat{\rho}] \leq 2n+1. \tag{37}$$

Optimal states must be quantum non-Gaussian

States which reach the upper bound (37) for $n > 0$ must necessarily be quantum non-Gaussian, i.e., they cannot be written as a mixture of Gaussian states (see e.g. Supplementary References [12, 13]).

To see this, notice that in (36), equality is reached if and only if the conditions $\langle\hat{X}\rangle_{\hat{\rho}} = \langle\hat{P}\rangle_{\hat{\rho}} = \text{Cov}(\hat{X},\hat{P})_{\hat{\rho}} = 0$ and $\Delta\hat{X}_{\hat{\rho}} = \Delta\hat{P}_{\hat{\rho}}$ are satisfied, or equivalently, $\langle\hat{a}\rangle_{\hat{\rho}} = \langle\hat{a}^\dagger\rangle_{\hat{\rho}} = \langle\hat{a}\hat{a}\rangle_{\hat{\rho}} = \langle\hat{a}^\dagger\hat{a}^\dagger\rangle_{\hat{\rho}} = 0$. These conditions can only be satisfied by a Gaussian state when $n = 0$, i.e., the vacuum state $|0\rangle$. The above statement then follows together with the convexity of the quantum Fisher information.

All pure states lead to equality in Supplementary Equation (33). In the case of a pure state, non-Gaussianity is equivalent to a negative Wigner function [14].

Hence, mixtures of Gaussian states will always perform sub-optimally for the estimation of a displacement amplitude in a "worst-case" scenario.

Average sensitivity bound

The same bound also holds for the average of Supplementary Eq. (32). We obtain

$$\mathfrak{F}_{\mathrm{mean}}[\hat{\rho}] \leq (\Delta\hat{X})^2_{\hat{\rho}} + (\Delta\hat{P})^2_{\hat{\rho}} \leq 2n+1, \tag{38}$$

and equality is reached only by states that are not displaced: $\langle\hat{X}\rangle_{\hat{\rho}} = \langle\hat{P}\rangle_{\hat{\rho}} = 0$.

Optimality of Fock states

The bounds given by Supplementary Eq. (37) and Supplementary Eq. (38) are saturated by Fock states $|n\rangle = (\hat{a}^\dagger)^n/\sqrt{n!}|0\rangle$. Indeed, one easily confirms that Fock states satisfy all of the optimality conditions for the two bounds given by Supplementary Eq. (37) and Supplementary Eq. (38), as stated above. Specifically, one obtains $(\Delta\hat{X})^2_{|n\rangle} = \langle n|\hat{X}^2|n\rangle = \frac{1}{2}\langle n|(\hat{a}+\hat{a}^\dagger)^2|n\rangle = n+\frac{1}{2}$, and similarly for $(\Delta\hat{P})^2_{|n\rangle}$, as well as $\mathrm{Cov}(\hat{X},\hat{P})_{|n\rangle} = 0$. This yields the covariance matrix

$$\Gamma_{|n\rangle} = \begin{pmatrix} n+\frac{1}{2} & 0 \\ 0 & n+\frac{1}{2} \end{pmatrix}, \tag{39}$$

with the two-fold degenerate eigenvalue $n+1/2$ (the degeneracy expresses the fact that the Fock state is equally sensitive in all directions). Since $\mathfrak{F}_Q[|\Psi\rangle, \hat{R}(\phi_{\mathrm{LO}})] = 4(\Delta\hat{R}(\phi_{\mathrm{LO}}))^2_{|\Psi\rangle}$ for all pure states, we find the exact equality

$$\mathfrak{F}_{\min}[|n\rangle] = 2n+1. \tag{40}$$

We obtain the same result for the mean value (32), since for pure states, the equality

$$\mathfrak{F}_{\mathrm{mean}}[|\Psi\rangle] = \mathrm{Tr}\Gamma_{|\Psi\rangle}, \tag{41}$$

holds. For the Fock state $|n\rangle$ this leads to $\mathfrak{F}_{\mathrm{mean}}[|n\rangle] = 2n+1$.

In summary, for a fixed energy (given by n), the Fock state provides the optimal precision for displacement detections with unknown phase. This is true for both strategies, i.e., preparing for the worst-case scenario (31) or optimizing the mean performance (32).

Supplementary Note 5 Classical limit

Based on the Glauber-Sudarshan P-representation,

$$\hat{\rho} = \int d\alpha P(\alpha)|\alpha\rangle\langle\alpha|, \tag{42}$$

we define classical states $\hat{\rho}_{\mathrm{cl}}$ as those for which $P(\alpha)$ describes a probability distribution. Here $|\alpha\rangle = \hat{D}(\alpha)|0\rangle$ is a coherent state. The classical limit is then given as the maximum quantum Fisher information, taken over all classical states $\hat{\rho}_c$. Since the Fisher information is convex, the maximum is attained by a pure coherent state $|\alpha\rangle$. Using $\mathfrak{F}_Q[|\alpha\rangle, \hat{R}(\phi_{\mathrm{LO}})] = 4(\Delta\hat{R}(\phi_{\mathrm{LO}}))^2_{|\alpha\rangle}$, we obtain

$$\mathfrak{F}_Q^{(\mathrm{cl})}[\hat{R}(\phi_{\mathrm{LO}})] := \max_{\hat{\rho}_{\mathrm{cl}}} \mathfrak{F}_Q[\hat{\rho}_{\mathrm{cl}}, \hat{R}(\phi_{\mathrm{LO}})] = 1, \tag{43}$$

which is independent of ϕ and α and corresponds to the sensitivity of the vacuum state. Thus, any observation of

$$\mathfrak{F}_Q[\hat{\rho}, \hat{R}(\phi_{\mathrm{LO}})] > 1, \tag{44}$$

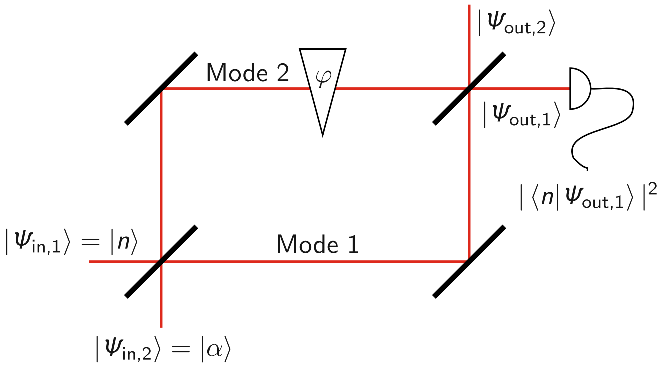

Supplementary Figure 3: **Analogy with a two-mode interferometer.** The presented measurements can be understood in terms of a two-mode interferometer, with a coherent state and a Fock state at the input ports $|\psi_{\text{in}}\rangle = |n\rangle|\alpha\rangle$. The output state is $|\Psi_{\text{out}}\rangle = \hat{U}(\varphi)|\Psi_{\text{in}}\rangle$, with $\hat{U}$ given in Eq. 49

reveals that the state $\hat{\rho}$ is non-classical according to the above definition [15]. This form of non-classicality is a necessary resource to overcome the classical limit eq. (43). The resulting upper bound on the Fisher information for amplitude measurements using classical states is given by

$$\mathcal{F}_Q = 4. \tag{45}$$

The corresponding bound for the phase measurement can be infered from

$$\mathcal{F}_Q(\delta) = \mathcal{F}_Q(\alpha)\left(\frac{\mathrm{d}(\delta\alpha)}{\mathrm{d}\alpha}\right)^2 . \tag{46}$$

Assuming that the detuning δ is small compared to the displacement Ω rate, we get

$$\tilde{\alpha} = \Omega t_{\mathrm{F}}\left(T + t_{\mathrm{F}}\right)\delta + \mathcal{O}(\delta^3) \tag{47}$$

and for the Quantum Fisher information

$$\mathcal{F}_Q(\delta) = \left(2|\alpha|\left(T + t_{\mathrm{F}}\right)\right)^2 , \tag{48}$$

which results in the SQL limit given in eq. (3) in the main text.

Supplementary Note 6 Analogy with a two-mode interferometer

Fock-state metrology can be understood in the wider context of a two-mode Mach-Zehnder interferometer [16] as shown in Supplementary Fig. 3. The evolution in a two-mode interferometer (with modes $\hat{a}$ and $\hat{b}$) is described by,

$$\hat{U}(\varphi) = \exp\left(-i\hat{J}_y\varphi\right), \tag{49}$$

where $\hat{J}_y = (\hat{a}^\dagger\hat{b} - \hat{b}^\dagger\hat{a})/2i$ and φ is the phase shift. For the two-mode transformation (49) one obtains the classical limit, i.e., the maximum sensitivity for two-mode classical states,

$$\mathfrak{F}_Q^{(\mathrm{cl})}[\hat{J}_y] = n_a + n_b, \tag{50}$$

where $n_a + n_b$ is the total number of excitations ($n_a = \mathrm{Tr}\{\hat{\rho}\hat{a}^\dagger\hat{a}\}$ and $n_b = \mathrm{Tr}\{\hat{\rho}\hat{b}^\dagger\hat{b}\}$). This bound is known as the shot-noise limit and coincides with the sensitivity bound for states that are separable among particles [17]. For a Fock state $|n\rangle$ in input mode $\hat{a}$, the quantum Fisher information reads

$$\mathfrak{F}[|n\rangle\langle n| \otimes \hat{\rho}_b, \hat{J}_y] = 2n_b n + n_b + n, \tag{51}$$

which yields a quantum-enhanced sensitivity for any $n > 0$ [16], in agreement with the results from the previous section.

The specific case of the displacement discussed in Supplementary Note 4 is recovered in the homodyne limit, in which a classical, highly populated coherent state is inserted in one of the input ports. We thus assume that input mode $\hat{b}$ is prepared in a coherent state $\hat{\rho}_b = |\alpha_0\rangle\langle\alpha_0|$ with $|\alpha_0|^2 = n_0 \gg 1$ particles, while the quantum state $\hat{\rho}_a$ of mode $\hat{a}$ input is arbitrary. Neglecting quantum fluctuations, by making the replacement $\hat{b} \to \alpha$ and $\hat{b}^\dagger \to \alpha^*$ in Eq. (49), we obtain an effective transformation of mode $\hat{a}$, described by

$$\hat{U}(\theta) = \exp\left(-(\alpha_0\hat{a}^\dagger - \alpha_0^*\hat{a})\varphi/2\right). \tag{52}$$

The evolution of mode $\hat{a}$ is effectively given by the displacement (29) if we rescale the parameter by $\alpha = -\alpha_0\varphi/2$. The precision of an estimation of the phase shift ϕ in the two-mode Mach-Zehnder interferometer is bounded by $\mathfrak{F}_Q[\hat{\rho}, \hat{H}(\phi_{\mathrm{LO}})]$, where $\hat{H}(\phi_{\mathrm{LO}}) = -\sqrt{n_0}\hat{R}(\phi_{\mathrm{LO}})$. The precision limits can be linked to the single-mode results from Methods section "Quantum metrology" and Supplementary Note 5 by a simple rescaling transformation: $\mathfrak{F}_Q[\hat{\rho}, \hat{H}(\phi_{\mathrm{LO}})] = n_0\mathfrak{F}_Q[\hat{\rho}, \hat{R}(\phi_{\mathrm{LO}})]$. Hence, the non-classicality bound for the Mach-Zehnder interferometer in the homodyne limit can be obtained from Eq. (43) as $\mathfrak{F}_Q^{(\mathrm{cl})}[\hat{H}(\phi_{\mathrm{LO}})] = n_0$. For a Fock state in the second input port, we obtain a precision of $\mathfrak{F}_Q[||n\rangle, \hat{H}(\phi_{\mathrm{LO}})] = 2n_0 n + n_0$, independently of the phase of α_0, which allows for sub-shot-noise sensitivity for $n > 0$, in agreement with our previous considerations (see Supplementary Note 4).

As discussed above, the classical bound and the Fock-state sensitivity can be equivalently obtained from the two-mode results (50) and (51), remembering that in the homodyne limit considered here, the contribution of n_a or n to the total number of particles is negligible due to $n_b = n_0 \gg n_a, n$.

Supplementary References

[1] Biercuk, M. J., Uys, H., Britton, J. W., VanDevender, A. P. & Bollinger, J. J. Ultrasensitive detection of force and displacement using trapped ions. Nature Nanotechnology **5**, 646–650 (2010). URL `http://www.nature.com/nnano/journal/v5/n9/full/nnano.2010.165.html`.

[2] Shaniv, R. & Ozeri, R. Quantum lock-in force sensing using optical clock Doppler velocimetry. Nature Communications **8**, 14157 (2017). URL `http://www.ncbi.nlm.nih.gov/pmc/articles/PMC5309847/`.

[3] Brownnutt, M., Kumph, M., Rabl, P. & Blatt, R. Ion-trap measurements of electric-field noise near surfaces. Reviews of Modern Physics **87**, 1419–1482 (2015). URL `http://link.aps.org/doi/10.1103/RevModPhys.87.1419`.

[4] Wineland, D. et al. Experimental primer on the trapped ion quantum computer. Fortschritte der Physik **46**, 363–390 (1998).

[5] Maiwald, R. et al. Stylus ion trap for enhanced access and sensing. Nature Physics **5**, 551–554 (2009). URL `http://www.nature.com/doifinder/10.1038/nphys1311`.

[6] Leopold, T. et al. A cryogenic radio-frequency ion trap for quantum logic spectroscopy of highly charged ions. arXiv:1901.03082 (2019). URL `http://arxiv.org/abs/1901.03082`. ArXiv: 1901.03082.

[7] Lakhmanskiy, K. et al. Observation of superconductivity and surface noise using a single trapped ion as a field probe. Physical Review A **99**, 023405 (2019). URL `https://link.aps.org/doi/10.1103/PhysRevA.99.023405`.

[8] Casella, G. & Berger, R. L. Methods of evaluating estimators. In Statistical Inference, 331 (Duxbury Press, Australia ; Pacific Grove, CA, 2001), 2nd edition edn.

[9] Roos, C. F. Ion trap quantum gates with amplitude-modulated laser beams. New Journal of Physics **10**, 013002 (2008).

[10] Pezzè, L., Smerzi, A., Oberthaler, M. K., Schmied, R. & Treutlein, P. Quantum metrology with nonclassical states of atomic ensembles. Rev. Mod. Phys. **90**, 035005 (2018). URL `https://link.aps.org/doi/10.1103/RevModPhys.90.035005`.

[11] Braunstein, S. L. & Caves, C. M. Statistical distance and the geometry of quantum states. Physical Review Letters **72**, 3439–3443 (1994). URL `http://link.aps.org/doi/10.1103/PhysRevLett.72.3439`.

[12] Filip, R. & Mišta, L. Detecting Quantum States with a Positive Wigner Function beyond Mixtures of Gaussian States. Physical Review Letters **106**, 200401 (2011). URL `https://link.aps.org/doi/10.1103/PhysRevLett.106.200401`.

[13] Genoni, M. G. et al. Detecting quantum non-Gaussianity via the Wigner function. Physical Review A **87**, 062104 (2013). URL `https://link.aps.org/doi/10.1103/PhysRevA.87.062104`.

[14] Hudson, R. When is the wigner quasi-probability density non-negative? Reports on Mathematical Physics **6**, 249–252 (1974). URL `http://linkinghub.elsevier.com/retrieve/pii/003448777490007X`.

[15] Rivas, A. & Luis, A. Precision quantum metrology and nonclassicality in linear and nonlinear detection schemes. Phys. Rev. Lett. **105**, 010403 (2010). URL `https://link.aps.org/doi/10.1103/PhysRevLett.105.010403`.

[16] Pezzè, L. & Smerzi, A. Ultrasensitive Two-Mode Interferometry with Single-Mode Number Squeezing. Physical Review Letters **110**, 163604 (2013). URL `https://link.aps.org/doi/10.1103/PhysRevLett.110.163604`.

[17] Pezzè, L. & Smerzi, A. Entanglement, Nonlinear Dynamics, and the Heisenberg Limit. Physical Review Letters **102**, 100401 (2009). URL `http://link.aps.org/doi/10.1103/PhysRevLett.102.100401`.
