# Supplementary material for: Motional Fock states for quantum-enhanced amplitude and phase measurements with trapped ions
Source: Nat Commun. 2019 Jul 2;10:2929. doi: 10.1038/s41467-019-10576-4 (PMC6606596; doi:10.1038/s41467-019-10576-4)
Supplement: Supplementary file 2 — Description of Additional Supplementary Files [file 41467_2019_10576_MOESM2_ESM.pdf]

Description of Additional Supplementary Files

File name: Supplementary Movie 1

Description: Fock state 0 metrology. Left panel: Wigner functions of original and displaced Fock state together with their product. Right panel: state overlap between original and displaced Fock state.

File name: Supplementary Movie 2

Description: Fock state 1 metrology. Left panel: Wigner functions of original and displaced Fock state together with their product. Right panel: state overlap between original and displaced Fock state.

File name: Supplementary Movie 3

Description: Fock state 2 metrology. Left panel: Wigner functions of original and displaced Fock state together with their product. Right panel: state overlap between original and displaced Fock state.
